# Response to “The History of Uterus Transplantation, Rewritten.”

**DOI:** 10.1097/AS9.0000000000000189

**Published:** 2022-08-02

**Authors:** Omer Ozkan, Nasuh Utku Dogan, Ozlenen Ozkan

**Affiliations:** From the * Department of Plastic Surgery, Akdeniz University Faculty of Medicine, Antalya, Turkey; † Department of Gynecology, Akdeniz University Faculty of Medicine, Antalya, Turkey.

## Dear Editors,

We read with great interest the editorial written by Testa et al.^1^ First of all, we would like to thank for their comment about our case as being the first successful uterus transplantation in the history. In this report and previous articles written by our team, we explained in detail the clinical aspects, surgical details and also ethical issues regarding the first successful uterus transplantation.^2–4^ Moreover, besides reporting the outcome of the first successful uterus transplant, we presented an exit strategy, which was one of the main subjects of the article.^2^ In our opinion, the technique presented in our case could be an option for recipients with previous miscarriages before performing hysterectomy. Regarding the historical aspects of the procedure, the attempt by Fageeh et al^5^ should be cited as the first uterus transplant. Although not successful, this case was scientifically published and led a step through the realistic struggle for the following cases. The case performed in 1936 in Germany ended with tragic death of the recipient because of infection caused by rejection of the uterus. As a catastrophic case, this operation had never been published.

The preparatory phase of uterus transplant is essential and requires a significant experience including not only animal studies but also complicated transplant surgeries, cadaveric dissections, and detailed knowledge of immunosuppressive agents. Our team involved in composite tissue transplants for more than 2 decades including extremity and face transplants, which all led us to perform the first uterus transplant with success both technically and functionally. Our team also pioneered high variety of composite tissue transplants and also one of the few centers in the world performing research and active surgery including not only uterus but also numerous composite tissue transplants (ie, double hand and face transplants).^6,7^ We greatly appreciate the scientific work of the Swedish team including studies in animal models but one should not deny the catalyzer effect of the first successful uterus transplant, which promoted the uterus transplant from merely animal models into clinical practice. It took more than 12 years from the beginning of the studies in animal models to the birth of first baby, and in our opinion, our success accelerated the studies of uterus transplantation, which, before our attempt, was only a concept on animal models.

**FIGURE 1.**
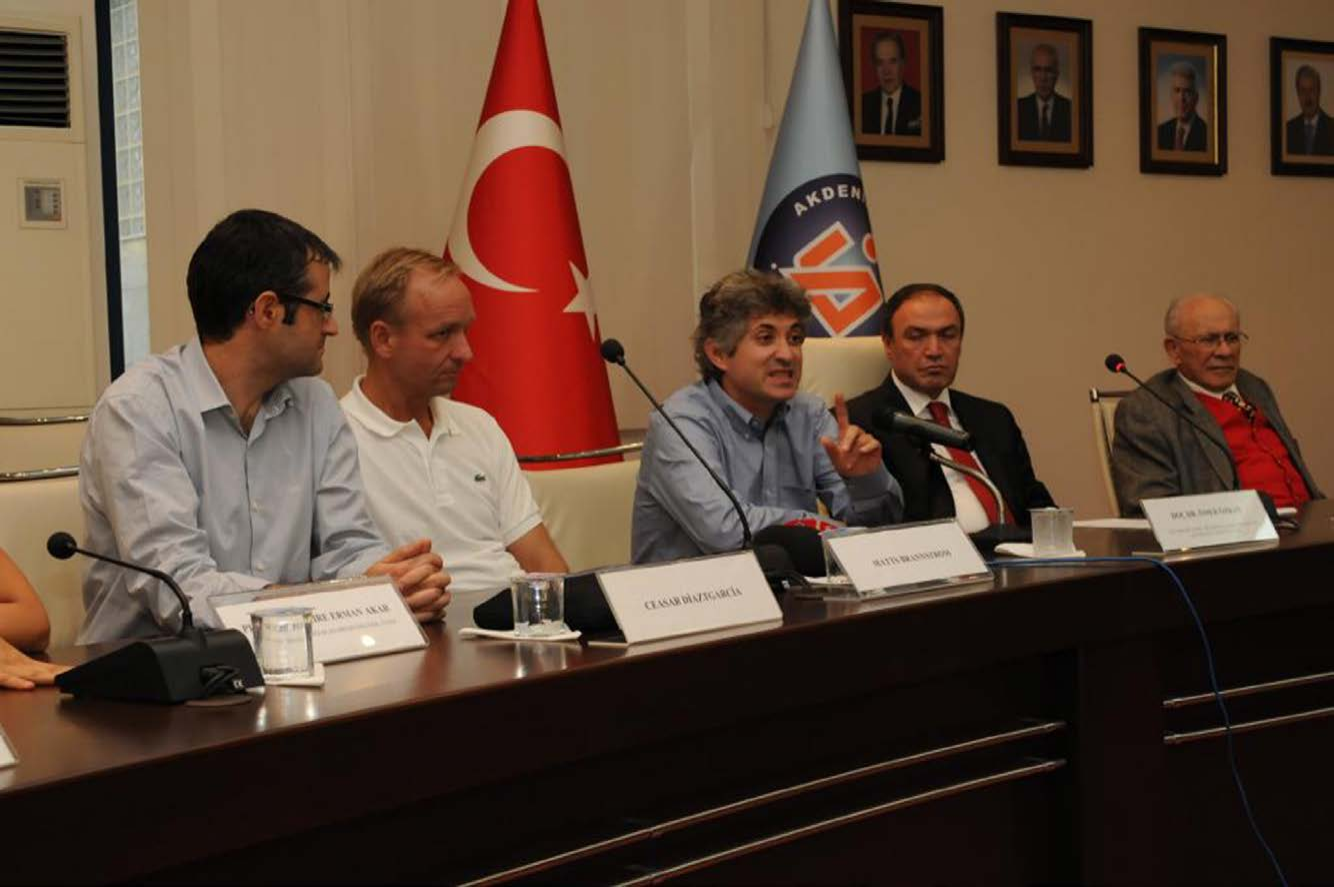
A photo taken from a press conference during the visit of Swedish team to Akdeniz University Department of Plastic Surgery, 10 days after the world first successful uterus transplant.

After the announcement of the first successful uterus transplant in Turkey in international media, the Swedish team contacted us and requested a visit to our clinic. Ten days after the transplant surgery, we were glad to welcome Swedish team in our clinic in Akdeniz University and they expressed their appreciation about passing one of the milestones of uterus transplant surgery. They noted carefully each and every detail of the surgery, and we were more than welcome to share all our surgical and immunologic experiences (Fig. 1). We were also proud to express that the details of our surgical techniques and immunosuppressive protocol was adapted by Swedish team in their surgeries.

Regarding ethical issues, every and each step of the procedures performed was discussed in detail both with the recipient and also with the professional teams including ethical committee, institutional review board, and Turkish Ministry of Health. All fundamentals of clinical ethics, as suggested by Testa et al,^1^ were fulfilled and clearly shared with all parties. None of the concepts suggested by Testa et al^1^ were violated including therapeutic misconception, autonomy, informed consent, and nonmaleficence. Indeed, in the course of the follow-up, hysterectomy was presented as a viable option and strongly suggested in order not to expose recipient to nephrotoxic effects of immunosuppression but instead, she opted to continue which should be respected in order not to violate autonomy of the recipient.^2^ The authors claimed us to take advantage of the recipient’s desire to have a baby and to be responsible for therapeutic misconception and also asked us how we properly informed our recipient regarding a procedure that has only been attempted twice before in history, without any success. Depending on these claims, one could also ask the logic, rationale and ethical issues behind the decision to continue after the first 3 failed attempts of Dallas team and also inclusion of 10 recipients instead of one in the trial performed by Swedish team.^8^ According to Testa et al,^1^ these could all be examples of violation of nonmaleficence, but actually they are not. Our brave struggle contributed to the development of successful uterus transplants and opened a new era in reproductive medicine.

Regarding the number of in vitro fertilisation (IVF) cycles and embryo transfer attempts, scientifically, there is no upper limit of IVF cycles and embryo Transfer (ET) before ceasing these attempts.^9–11^ Of course, all these depend on the motivation and general health condition of the patient. Similarly, one of the recipients in the first trial of Swedish group experienced 6 miscarriages, we do not know the details of the IVF procedures nor the number of ET attempts.^12,13^ Moreover, there are also other recipients in Czech group with repeated miscarriages.^14^ The maximum number of ET that could be performed in case of recurrent IVF failures is obscure.

Regarding relatively long period of keeping the graft, the doses of the immunosuppressive drugs used in uterus transplant patients are quite lower than the doses used in other composite tissue transplants. Kidney functions of the recipient were strictly controlled all through the prepregnancy period and in antenatal follow-up as well. It has been more than 11 years since the uterus transplantation of our case but the kidney function (including kidney function test and glomerular filtration rate) were all within normal limits, including the last control. This was the first successful case of uterus transplant without any preceding index case. There are some gray zones that all should be evaluated on patient basis such as inclusion of a recipient with a history of cancer (eg, cervical cancer history) or permission of second birth jeopardizing the recipient and bringing extra load on kidney functions. Moreover, in the first trial of Swedish team there were 2 women who were followed for 81 and 77 months, respectively, before undergoing hysterectomy. The first case experienced multiple miscarriages without a live birth.^13^ As in these 2 cases of Swedish team, the long period of keeping the graft should be evaluated in this context.

Finally, any health profession, somehow associated with transplant medicine should respect the heroic struggle and honorable memory of Professor Thomas E. Starzl, who did not only open the door to hepatic transplants but also saved lives of thousands of people all around the world and also created a new field of medicine as immunology. We are very glad to be cited in the same context together with Professor Starzl.

In conclusion, uterus transplant is ever growing field of medicine with a high media and public interest. In our opinion, every negative and positive aspects and experiences about this issue should be discussed scientifically without any prejudice and scientific bias for the sake of the women desiring to have their genetic babies. We would like to thank once again Testa et al^1^ for their nice editorial on our case and hope that all these scientific discussion contribute to the improvement of uterus transplant concept that was just a dream a decade ago.
